# Incorporating sex, gender and vulnerable populations in a large multisite health research programme: The Ontario Pharmacy Evidence Network as a case study

**DOI:** 10.1186/s12961-017-0182-z

**Published:** 2017-03-20

**Authors:** Martin Cooke, Nancy Waite, Katie Cook, Emily Milne, Feng Chang, Lisa McCarthy, Beth Sproule

**Affiliations:** 1 Department of Sociology and Legal Studies, University of Waterloo, 200 University Drive W, Waterloo, ON N2L 3G1 Canada; 2 School of Public Health and Health Systems, University of Waterloo, 200 University Drive W, Waterloo, ON N2L 3G1 Canada; 3 School of Pharmacy, University of Waterloo, 200 University Drive W, Waterloo, ON N2L 3G1 Canada; 4 Psychology Department, Wilfrid Laurier University, 75 University Avenue West, Waterloo, ON N2L 3C5 Canada; 5 Department of Sociology, MacEwan University, 10700 104th Ave NW, Edmonton, AB T5J 4S2 Canada; 6 Women’s College Research Institute at Women’s College Hospital, 76 Grenville St., Toronto, ON M5S 1B2 Canada; 7 Leslie Dan Faculty of Pharmacy, University of Toronto, 144 College St., Toronto, ON M5S 3M2 Canada; 8 Department of Family and Community Medicine, Faculty of Medicine, University of Toronto, 500 University Ave., Toronto, ON M5G 1V7 Canada; 9 Centre for Addiction and Mental Health, 101 Queen St. W., Toronto, ON M6J 1H4 Canada

**Keywords:** Sex, Gender, Pharmacy practice research, Research collaboration

## Abstract

**Background:**

Funders now frequently require that sex and gender be considered in research programmes, but provide little guidance about how this can be accomplished, especially in large research programmes. The purpose of this study is to present and evaluate a model for promoting sex- and gender-based analysis (SGBA) in a large health service research programme, the Ontario Pharmacy Evidence Network (OPEN).

**Methods:**

A mixed method study incorporating (1) team members’ critical reflection, (2) surveys (n = 37) and interviews (n = 23) at programme midpoint, and (3) an end-of-study survey in 2016 with OPEN research project teams (n = 6).

**Results:**

Incorporating gender and vulnerable populations (GVP) as a cross-cutting theme, with a dedicated team and resources to promote GVP research across the programme, was effective and well received. Team members felt their knowledge was improved, and the programme produced several sex- and gender-related research outputs. Not all resources were well used, however, and better communication of the purposes and roles of the team could increase effectiveness.

**Conclusions:**

The experience of OPEN suggests that dedicating resources for sex and gender research can be effective in promoting SGBA research, but that research programmes should also focus on communicating the importance of SGBA to their members.

**Electronic supplementary material:**

The online version of this article (doi:10.1186/s12961-017-0182-z) contains supplementary material, which is available to authorized users.

## Background

Over the past three decades, several important developments in health and social science literatures have affected the way that differences between men and women are addressed across fields of health research and policy. These changes are not independent, but reflect a more general shift toward consideration of the social as well as the biological dimensions of these differences and their causes, and toward including constructionist perspectives on social inequalities and differences alongside positivist explanations. Together, these changes have led research funders in Canada and elsewhere to require that research teams incorporate these perspectives in their research plans. This is the challenge that this paper hopes to help address.

An important change that has taken place across health and biomedical sciences has been the recognition that differences between males and females have been systematically ignored in health research. This has included women being under-represented in research designs, or absent entirely, resulting in results being inappropriately generalised from male-only samples to the entire population [1]. Evidence remains of continuing under-representation of women in research from the United States of America and other countries. This includes a lack of reporting of male/female differences, despite the United States National Institutes of Health requiring, since 1993, that federally supported Phase III clinical trials include female participants [2, 3].

Over the past several decades, social causes have attracted greater attention as explanations for observed differences between males and females for a range of health-related outcomes and processes. Since the 1970s, researchers have increasingly referred to the role of ‘gender’, or the socially derived differences between women and men, boys and girls, along with the physiological differences associated with ‘sex-linked biology’ [4]. Although the biological and social factors may be difficult to conceptually and methodologically delineate, this has led to greater attention to the importance of aspects of culture, norms and social roles, and their implications for a wide range of patient diagnoses, treatments and health outcomes [5–7]. Researchers concerned with understanding gender differences in health have also called for attention to the role of unequal social power in their creation and maintenance. Drawing from critical feminist studies, this includes consideration of how the epistemology and practice of health and medical research might themselves be shaped by gender [8].

Other insights from critical social science have shaped the study of gender and health. Developed in the 1980s and 1990s, ‘intersectional’ approaches see social dimensions of inequality, such as gender, race/ethnicity, sexuality and social class, not as separate but as mutually constitutive and interlocking, whereby the implications of one’s positon on one dimension cannot be fully understood without reference to others [9]. The field of queer studies has contributed the ideas that both sexuality and gender identities are fluid and multiple, rather than fixed and binary. This has led to a new focus on the health of transgendered people, as well as lesbian, gay, bisexual and other sexual minorities, and of the importance of including these dimensions in health research and data [10, 11].

In response to the recognition of the complexities of sex-linked biology and gender, and their critical importance for health services research and policy, there have been calls for assessments of the scientific, social and economic benefits of research to be made specifically with an eye to reducing gender bias [1]. To this end, funding agencies have required researchers to incorporate both sex and gender into their research. Approaches to considering both the social and biologically-linked dimensions throughout research projects are sometimes referred to as ‘sex- and gender-based analyses’ or SGBA [12]. SGBA has been made either a requirement or a priority by public research funders [13–15]. Similar to the National Institutes of Health [16], Health Canada implemented a Sex and Gender-Based Analysis Policy in 2000 [12], and applicants to any of the Canadian Institutes of Health Research (CIHR) funding programmes are now expected to incorporate sex and/or gender in research designs or to justify why they are not relevant [15, 17]. As that federal health research funding agency has put it, “*if our research designs do not take sex and gender into account, the evidence we generate may be incomplete or simply incorrect; we risk not only doing harm (such as extrapolating findings based on male samples to females), but also missing critical opportunities to improve health (for example, not detecting the benefits of an intervention in a subgroup of men)*” [18].

The proportion of CIHR-funded research projects incorporating sex and gender rose from 28% in 2010 to 48% in 2011, a result partly attributable to this new emphasis [19]. However, barriers to the integration of sex and gender into health research projects remain. Day et al. [20] identified a lack of consistent terminology, challenges with applying the concepts of sex and gender and using them to interpret results, and a lack of appropriate indicators on datasets as key challenges. Despite the priority given to integrating sex and gender in health research, there is also evidence that funding agencies have not given a great deal of practical guidance about how these dimensions should be integrated in research, instead leaving researchers the freedom and responsibility for developing their own plans and models. Several agencies and research groups have provided case studies of SGBA approaches within specific health research and healthcare settings [21–28]. Examples include *What a Difference Sex and Gender Make: A Gender, Sex and Health Research Casebook* [18] and *Rising to the Challenge: Sex- and Gender-Based Analysis for Health Planning, Policy and Research in Canada* [29]. Researchers have also developed sex- and gender-specific tools in order to advance research and methods in this area. Doull et al. [30] have developed the *Sex and Gender Appraisal Tool* to assess systematic reviews with reference to sex and gender issues, and *Briefing Notes* [31] to aid researchers to implement sex/gender analysis in systematic reviews. Oertelt-Prigione et al. [6] have established GenderMedDB, an archive of medical literature containing sex- and/or gender-specific analyses.

Most of these examples focus on application of SGBA to specific research questions, within discrete research projects. In such projects, typically having one main source of data and a single research team, one might expect that researchers could easily determine how sex and gender might be important, and how to include these dimensions in the research design. For large and multi-centre research programmes, the intentional and systemic incorporation of sex and gender poses a greater challenge. There may be multiple sub-projects, and research questions and approaches might be emergent over the course of a programme. The extant literature provides few examples relevant to integrating a SGBA model in these larger organisations. This paper aims to begin to fill this gap by describing how considerations of sex, gender and vulnerable populations (GVPs) were promoted within a large multisite health research programme, reporting the results of an evaluation of the model and the lessons learned in its implementation.

## Methods

### The multisite research programme

The Ontario Pharmacy Evidence Network (OPEN) (www.open-pharmacy-research.ca) is a multi-institutional and multidisciplinary research programme established to produce policy-relevant research in the context of changes to medication management services in the province of Ontario, Canada. It has received several sources of grant support including its largest grant of CDN$5.7 million from the Ontario Ministry of Health and Long-Term Care’s (MOHLTC’s) Health Systems Research Fund (2013–2016). At the time of writing, the collaboration includes over 31 investigators, 24 staff and more than 60 students from several disciplines at 10 institutions. At inception, it included six main research projects, each of which involved a number of discrete studies: Pharmacist Services Evaluation Framework, MedsCheck and Pharmaceutical Opinion, Pharmacists as Immunizers, Pharmacist Prescribing, Deprescribing Guidelines for the Elderly, and Chronic Pain Management by Rural and Urban Community Pharmacists. Each of these projects had an interdisciplinary team (hereinafter ‘project teams’) responsible for defining their research agendas, conducting the research and leading dissemination and knowledge translation activities. An additional 18 research studies were initiated through an “Applied Health Research Questions” process, in which health system policymakers and service providers could address specific research questions to the OPEN teams.

Similar to other large research funding agencies, MOHLTC requires plans for addressing sex and gender to be included in research funding applications [32]. It was also important to the OPEN leadership that its research projects incorporate a range of other dimensions of vulnerability (e.g. race/ethnicity, age, socioeconomic status, dis/ability), along with sex and gender. Our goals, conceived very broadly at the project’s outset, were (1) to increase knowledge and awareness of both sex and gender among OPEN research team members and (2) to increase the integration of sex, gender and intersecting dimensions of social inequity in OPEN research projects. To do that, we proposed and implemented a model in which GVPs were incorporated as a cross-cutting theme, supported by a GVP team and dedicated resources.

### The GVP team model

**Fig. 1:**
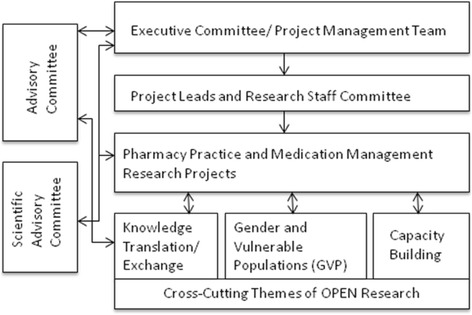
OPEN organisational structure

GVP was positioned as one of three cross-cutting themes that supported and provided input into OPEN research projects (the other two were Knowledge Translation and Exchange, and Capacity Building, see Fig. 1). These themes were given the same importance in the OPEN organisation as the six project teams, and were composed of researchers, administrative staff and trainees. Like other OPEN teams, the GVP team controlled their own budget, participated in OPEN activities, meetings and reporting, and conducted their own research.

Each of the other project teams had at least one representative on the GVP team, and several GVP team members were leads of their project teams. GVP members were also selected from among OPEN members based on their interest and previous experience with research related to sex and gender and social inequality. The GVP team had two co-leads; one was a non-pharmacist social scientist with expertise in health inequities and health research in vulnerable populations, and the other was one of the pharmacist co-leads of the OPEN programme, included to signal the importance of GVP activities within the OPEN research programme and to coordinate activities across project teams. A part-time non-pharmacist research coordinator with graduate training in social science research was hired to keep track of GVP sub-projects and budgets, schedule meetings, coordinate communication, and assist with research and writing.

The GVP team had its own budget of approximately CDN$50,000 per year for 2013–2016. This was intended to pay for the salary of the part-time research coordinator, to provide a small amount for administrative supplies and to support additional research conducted by the GVP team. At the outset of the research programme, it was anticipated that the GVP team would see opportunities to augment the work of other project teams as the research progressed; we expected this could be conducting literature reviews, performing additional secondary analyses or collecting qualitative data to further explore GVP themes that might be uncovered by the projects, but which they had not planned and therefore might not have the capacity to do themselves.

With broad goals of increasing the attention paid to sex and gender, as well as to intersecting dimensions of vulnerability within OPEN, the GVP team undertook activities intended to support consideration of ‘GVP questions’ in all phases of OPEN research projects and to build capacity by educating OPEN members about sex, gender and vulnerable populations in medication management research. These activities included:

1. Consulting with individual OPEN project teams, both through formal inquiries and other more informal interactions (e.g. conversations at meetings, through involvement in other OPEN projects). These were intended to assess their activities and needs with regard to sex, gender and vulnerable populations research, and to identify opportunities to assist them.
2. Developing an online module using a web-based learning management system to provide background information as well as resources and tools, such as a video introducing concepts and frameworks for sex- and gender-based analysis and intersectionality.
3. Constructing recommended survey questions to be used by OPEN project teams collecting their own survey data related to sex, gender, sexual orientation, ethnicity, race, Aboriginality, and other dimensions of identity. Consideration was given to asking questions in a way that was inclusive, avoiding, for example, asking respondents’ gender in a way that requires them to choose only two options when neither might reflect their own identity, or to identify as ‘other’, potentially reinforcing a sense of marginalisation. At the same time, these questions would encourage standardised collection of these data across OPEN research projects, and comparability to some other national data sources. Proposed questions were supported with a background document that included references and other potential questions.
4. Adapting the MOHLTC’s Health Equity Impact Assessment (HEIA) tool [33] to medication management research. This tool provides a template and workbook that help users consider the impacts of programme or policies on health equity, directing users to specifically consider effects on a range of populations, including immigrants, Indigenous peoples, age-related groups, people with disability, as well as sex and gender. By adapting the tool for pharmacy practice researchers, we hoped that it would help our colleagues and others to consider how to incorporate these dimensions in their projects.
5. Identifying gaps related to sex and gender in the medication management and pharmacy practice literature and conducting their own research to fill these knowledge gaps.

### Evaluation methods

A mixed method evaluation of the OPEN GVP activities was conducted. Data sources included surveys and qualitative interviews of OPEN members 18 months after OPEN’s launch, an OPEN member end-of-grant survey, and GVP team meeting notes and members’ critical reflections [34, 35] with respect to the creation and implementation of the GVP team model. Data collection procedures were approved by institutional research ethics bodies at the University of Waterloo, McMaster University and University of Toronto. All identifying information was removed to ensure participant confidentiality.

### Interim OPEN member survey and interview

At the midpoint of funding in 2014, OPEN researchers, staff and students were surveyed and interviewed in order to understand the perceived impact of the GVP team on knowledge among the OPEN project teams as well as their use of GVP resources. Survey and interview questions asked about members’ experiences and perceptions of the operation of OPEN, including the role and effectiveness of the GVP Team (See Additional file 1 for questionnaire content). The survey was distributed to all current and past OPEN team members, using Qualtrics online survey software.

Participants in the qualitative interviews were recruited using a purposive sampling method to ensure appropriate representation of member roles and locations. To ensure respondent confidentiality, a consultant external to OPEN conducted the interviews, using a semi-structured questionnaire (Additional file 1). Interviews were conducted over the phone, using Skype or in-person, and were audio-recorded and transcribed verbatim. Transcripts were analysed thematically [36] with the use of NVivo 10 software. The analysis focused on understanding how participants understood and engaged with GVP team members, as well as their views of OPEN GVP initiatives and resources. The analysis took an inductive approach; one author coded and recoded segments of qualitative data into higher-level and refined themes with input from one of OPEN’s co-leads. Fifty-three OPEN researchers, staff and students were invited to participate. A total of 23 interviews were conducted, a number that the interviewer and the coders felt allowed them to achieve ‘saturation’, whereby there were no new themes emerging from additional interviews.

### End-of-study OPEN member survey

The GVP team conducted an end-of-study survey in January and February 2016. An online questionnaire was sent to members of each of the six project teams, asking them to identify projects and sub-projects conducted as part of the broader OPEN research programme. One member from each team completed the survey. For each sub-project, the questionnaire asked whether and how sex and gender had been incorporated, and whether and how the project team had made use of the resources created by the GVP team. The survey was administered using Qualtrics survey software. Survey responses were tabulated using descriptive statistics.

### Meeting notes and critical reflection

Starting in June 2013, members of the GVP team met bi-weekly, either in person or by teleconference. These meetings involved discussions about how the GVP team could best serve the project teams, how to communicate with them and what GVP activities would be most beneficial. Notes taken during these meetings were reviewed and key themes were identified and incorporated throughout this paper. Two critical reflection sessions with the GVP team co-leads and one session with the entire GVP team were held. These sessions focused on recounting experiences of developing, implementing and evaluating the model. Notes were recorded by one member of the GVP team and circulated to the group for comment, elaboration or revision.

## Results

### Midpoint OPEN member survey and interview

At the midpoint of the project, the GVP team had reached out to other project teams, both individually and at programme meetings, about the team’s availability to consult on GVP-related research. The online module and other resources had been made available and were promoted using the OPEN monthly newsletter.

Thirty-seven OPEN researchers, staff and students out of 85 eligible past and present OPEN members completed the midpoint survey (44% response rate). The survey included questions about which dimensions of gender or vulnerability were included in the research projects at the time and provide a snapshot of the GVP-related activities at midpoint, and the use of the GVP resources. Most respondents (90%) indicated that sex, gender or vulnerable groups were being considered within their projects’ research. These dimensions included biological sex (72%); elderly or youth (70%); gender (57%); rural/remote populations (41%); low-income people (38%); inner-urban populations (30%); immigrants (27%); ethno-racial minorities (19%); those with limited literacy/health literacy (19%); people with disabilities (14%); non-English linguistic communities (11%); sexual minorities (11%); gender diverse people (11%); Indigenous peoples (5%); and religious/faith communities (3%).

Respondents were asked to assess their own knowledge about sex, gender and vulnerable population research, thinking back to the beginning of the OPEN research programme and at the time of the survey (midpoint); 30% reported that they had ‘little’ knowledge and 5% reported that they had ‘high’ levels of GVP knowledge at the beginning of the project. By comparison, 14% reported that they had ‘little’ knowledge and 48% reported that they had ‘high’ levels of knowledge after their involvement in OPEN.

The uptake of GVP materials and supports varied across project teams. Almost half (46%) of respondents said that their teams had used the recommended survey questions; 19% had used the GVP suggested survey question rationale and background materials; 14% had individual consultations with the GVP team; 14% reported that they had considered GVP issues independently of the GVP team; and 11% had used the GVP sex- and gender-based analysis learning module. The main users of services were project leads (43%), followed by students (20%), collaborator/communication personnel (16%) and staff (11%).

When asked about their perceptions of the GVP team activities, 47% of survey respondents were satisfied with the GVP component of OPEN; 31% were ‘unsure’. When asked about how the GVP team resources and support could be improved, respondents reported the GVP team could provide more educational and background materials (32%), research design support (25%), data analysis support (19%), and individual consultations (8%).

The interview respondents commented on how the GVP team helped raise awareness around vulnerable populations and sex and gender differences, introduced OPEN members to new literature and the importance of inclusive survey design. These issues had previously been ‘not on the radar’ of some respondents. As one project team lead noted, “*this exposure through OPEN opened up my eyes to a whole area of research I didn’t know existed, and I wouldn’t have had that opportunity if it had not been for the GVP team*.” Among students, this educational opportunity was valued as relatively rare, as the consideration of vulnerable populations in research was not part of the pharmacy curriculum. Respondents appreciated how the GVP team activities ‘surfaced’ domains traditionally neglected in the field but important to ensuring quality medication management practice.

### Midpoint reflection and action

At the mid-period of the OPEN programme, the GVP team reflected on the survey and interview results and felt that, while respondents recognised the value of having representatives from the GVP team working ‘alongside’ them on their research teams, there was lack of clarity about the role of the GVP team. OPEN members also expressed uncertainty around which of the several dimensions of vulnerability mentioned in the GVP team’s materials were most important to consider in their research.

In response, the GVP team undertook several activities to clarify its role, activities and availability for consultation. This included re-stating the offer of assistance to project teams through the OPEN newsletter and in-person meetings with project teams. A list identifying dimensions of vulnerability that were ‘priorities’ for the project was created and circulated to the project teams. These were identified by drawing on various sources including the stated requirements of the Health Systems Research Fund, the mandate letters from the Premier to provincial ministers outlining priority areas for their departments, a scoping review on health disparity research in pharmacy practice conducted by members of the GVP team [37], and feedback from the OPEN Knowledge User and Scientific Advisory Committees. Priority dimensions included sex-linked biology, gender and gender identity, sexual orientation, age-related groups, rural communities, people with low income, newcomers to Canada, ethnic and racialised minorities, Indigenous peoples, people with mental illness, people with drug addictions, people with disabilities, linguistic communities, and people with limited literacy.

Through GVP team meeting discussions, interactions with the project teams and in searching for SGBA training material, it was apparent that little attention appeared to have been paid to SGBA in medication management or pharmacy practice research or research organisations. As a result, several research questions became important to the GVP team. In addition to conducting a scoping review of health inequalities in pharmacy research [37], the team initiated scoping reviews to understand how sex and gender had previously been incorporated in pharmacy practice research [38], and to examine the state of the literature regarding pharmacy services and Indigenous populations, as Aboriginality is an important dimension of health inequity in Ontario and Canada. Members of the GVP team also obtained seed funding for a separate project to examine geo-spatial patterns of community pharmacies and pharmacy services in relation to vulnerable populations.

### End-of-study OPEN member survey

The 2016 end-of-project survey of OPEN teams collected data on how the OPEN teams had used GVP resources, as well as how sex, gender and vulnerable populations had been included in their research activities; six out of six teams responded (100% response rate).

All six OPEN project teams reported using the GVP team’s recommended survey questions in their research. Four project teams had used the online sex, gender and intersectionality learning module. Two project teams reported using individual consultations with the GVP team, however, other project teams reported that arranging formal consultations with the GVP team was not necessary as they had a team member who was part of the GVP team and able to provide the necessary support. None of the OPEN teams mentioned using the modified HEIA tool in their research.

## Discussion

OPEN’s GVP model is one example of how sex and gender can be incorporated as a theme across a large research programme. The GVP model changed over the course of the OPEN research programme, in response to improved understanding about the work of the project teams and how best to promote the inclusion of dimensions of vulnerability in medication management research. There were some lessons learned throughout the process.

In the initial stages of the research, some of the OPEN project teams were unsure what questions to ask the GVP team or how to use GVP support. At the same time, the GVP team struggled with how to engage the project teams in a non-intrusive way, particularly at the busy, yet strategic, time early in the grant funding period. Part of this was due to a desire of the GVP team members to not be seen as pushing a sex and gender research agenda on the other project teams, members of which were well-respected leaders in pharmacy practice research with well-developed research goals. In retrospect, the GVP team could have been somewhat more active in communicating with the other teams. Over time, a balance developed whereby the GVP team positioned itself as available and supportive, but also found it effective to ‘jump start’ conversations, and proactively offer advice and examples. In particular, engaging in our own GVP-focused research provided the other project teams with examples of the kinds of approaches they could take or research questions they could ask within their own work. In the end, the members of the other teams were more receptive to social science perspectives than we perhaps had given them credit for. We learned that there was wider recognition of the importance of these dimensions than we had anticipated.

It was also clear that we might have given more careful thought to what, exactly, ‘vulnerability’ might mean in the context of pharmacy practice research. Again, our original intention was to not be prescriptive, but to try to support our colleagues in conducting research on whatever social dimensions they might find to be important. The use of the HEIA tool led to the GVP team considering a range of ‘vulnerable’ populations, without providing a general definition of vulnerability that would help OPEN teams consider which of these dimensions might be important for their research. The request from the OPEN community at project midpoint for a concrete list of vulnerabilities for the project to focus on was a signal that more specificity was required. We tried to be more systematic in our identification of vulnerable populations in our response but failed to provide much better guidance. Having a better definition of vulnerability in the context of pharmacy practice research and a shorter list of priority populations might have given us a better opportunity to provide specific resources related to those dimensions, and to help our colleagues think through how these dimensions might intersect with sex and gender in relation to their research topics.

We also learned the value of providing a budget dedicated to these activities. Having funds set aside for the GVP team was extremely useful and allowed the team to pursue its own focused research projects. At the outset, we were unsure where these funds would be needed the most and expected that some would be spent augmenting the research of other OPEN projects. In the end, the OPEN project teams already had full research agendas. They were not in a position to take advantage of these financial resources to pursue additional GVP-related questions. These resources were more productively used for GVP research that was led by the GVP team members and was independent of the other research projects.

Of course, there are limitations to this study. OPEN is composed of a particular team of researchers and, among them, those who agreed to participate in the survey and/or interview might be different from those who did not. The participants involved in this study may therefore not be representative of the larger population of pharmacy practice and health researchers. For example, the OPEN team might have been more receptive to considering issues related to sex, gender and vulnerable populations in their research than were other researchers, as they chose to join a group that made these issues an important theme. The results are illustrative of our experience within a particular setting, but may hold lessons that are applicable to others.

The OPEN research teams will be writing manuscripts based on their OPEN work for some time. Although we do not know what research would have been done by the programme in the absence of a GVP team, we are hopeful that many of these will include more thorough considerations of sex and gender and intersecting dimensions of vulnerability than would otherwise be the case. Follow-up over the next couple of years will be telling and provide further insight into the effectiveness of the GVP team.

Considerable interest has been generated in the issues of sex, gender and dimensions of vulnerability from within and beyond OPEN. Invitations to speak on topics related to what we have called ‘GVP’ have been received from the pharmacy practice community. In response to presentations, colleagues have expressed a commitment to conducting inclusive research and have indicated that they found resources, such as the inclusive survey questions, to be helpful. Similarly, our publications in this area have received positive social media commentary. We take this as evidence that the need for education and tools to support the integration of sex, gender and intersecting dimensions in research extends beyond the OPEN research community.

## Conclusion

Integrating considerations of sex, gender and intersecting dimensions of vulnerability into health research remains challenging, with barriers related to a lack of familiarity with some key concepts and a lack of tools to facilitate data collection and analysis [20]. The experience of OPEN suggests that an interdisciplinary cross-project structure can be a useful approach in the context of a large and complex research programme with several sub-projects and number of research priorities. We hope that our description of the model, and our successes and shortcomings, will help to inform the activities of other teams who are faced with the task of incorporating sex and gender in their research. We also encourage other researchers to share their own experiences, tools and approaches, in order to further develop ‘best practices’ for doing this work within large and multi-centre research programmes.

## Additional file

Additional file 1: Appendix. Interim OPEN Member Survey and Interview Guide. (DOC 82 kb)
