# Supplementary material for: Incorporating sex, gender and vulnerable populations in a large multisite health research programme: The Ontario Pharmacy Evidence Network as a case study
Source: Health Res Policy Syst. 2017 Mar 20;15:20. doi: 10.1186/s12961-017-0182-z (PMC5360067; doi:10.1186/s12961-017-0182-z)
Supplement: Additional file 1: Appendix. — Interim OPEN Member Survey and Interview Guide. (DOC 82 kb) [file 12961_2017_182_MOESM1_ESM.doc]

**Additional file 1**

**Appendix A. Interim OPEN Member Survey and Interview: Online Survey**

*Thinking of activities/ resources related to the Gender and Vulnerable Populations theme…*

1. Have you considered biological sex (male/ female) as a variable in your OPEN research initiatives?

- Yes
- No

1. Have you considered gender dynamics (particular experiences of men, women, girls, boys or gender diverse people) in your OPEN research initiatives?

- Yes
- No

1. Which of the following vulnerable populations are included in your OPEN research initiatives? Select all that apply:

- Aboriginal peoples
- Age-related groups (e.g. older adults, youth)
- People with disabilities
- Ethno-racial Communities
- Francophone Communities
- Linguistic Communities (non-English speaking)
- People with limited literacy/ health literacy
- Religious/Faith Communities
- Rural/remote populations
- Inner-urban populations
- Individuals with low socioeconomic status
- People without a family doctor
- Newcomers to Canada (i.e., immigrants, refugees, undocumented migrants, etc.)
- Sexual minorities (i.e., Gay, Lesbian, and Bi-sexual populations)
- Gender diverse people (i.e., Trans, Two-spirited, Queer, etc.)
- Other (any other relevant population group not captured above. Please specify _________________

1. Which materials developed by the GVP team have you referred to in designing or conducting your OPEN research (choose all that apply):
   - Sex/ Gender-based analysis learning module on LEARN
   - List of recommended survey questions that are inclusive of GVP groups
   - Suggested Survey Question Rationale Document
   - Health Equity Impact Assessment Tool
   - Individual consultation with GVP Team member(s)
   - Other (Please specify):
2. How can the GVP Team better support your research?

- More educational and background materials
- More research design support
- More data analysis support
- More individual consultations
- Other (Please specify):

1. What was your level of knowledge about issues related to gender and vulnerable populations in research….

|  | Not at all knowledgeable |  |  |  | Moderately knowledgeable |  |  |  | Extremely knowledgeable |
| --- | --- | --- | --- | --- | --- | --- | --- | --- | --- |
| When you began working with OPEN |  |  |  |  |  |  |  |  |  |
| How knowledgeable are you now |  |  |  |  |  |  |  |  |  |

1. Overall, how satisfied are you with the GVP component of OPEN?

|  | Very satisfied | Somewhat satisfied | Neutral | Somewhat dissatisfied | Very dissatisfied | Not sure |
| --- | --- | --- | --- | --- | --- | --- |
| Gender and Vulnerable Populations | ☐ | ☐ | ☐ | ☐ | ☐ | ☐ |

1. How would you like the GVP team to connect with you when they have news/ resources to share?

|  | Email | Phone | Newsletter | Via team lead | Via research staff | Other |
| --- | --- | --- | --- | --- | --- | --- |
| Gender and Vulnerable Populations | ☐ | ☐ | ☐ | ☐ | ☐ | ☐ |

Please provide a bit of detail on your “other” preferred method of communication: _________

***We have a few demographic questions for you.***

1. When did your involvement (employment, contribution, etc.) with OPEN begin?

Start month __ | Start year ____

1. Are you presently working with OPEN?

- Yes
- No
  1. When did you finish working with OPEN?

End month __ | End year ____

#### What is/was your primary role with OPEN?

- Project Lead
- Co-investigator
- Collaborator or Consultant
- Research coordinator
- Research assistant
- Management or Communication
- Student researcher (graduate or undergraduate)
- Postdoctoral position
- Other (please specify: _________)

#### What other roles do/did you have with OPEN? Select all that apply.

- Project Lead
- Co-investigator
- Collaborator or Consultant
- Research coordinator
- Research assistant
- Management or Communication
- Student researcher (graduate or undergraduate)
- Postdoctoral position
- Other (please specify: _________)
  1. [Students: What is/was your program of study?]
- BScPhm
- BScPhm Co-op
- PharmD
- MA
- PhD
- Other (please specify: _________)

1. At which OPEN site are you primarily affiliated

- Bruyere Research Institute/ University of Ottawa
- McMaster University
- University of Toronto
- University of Waterloo
- Western University

1. What is your educational background/training (select all that apply)?

- BScPharm
- Bachelor’s level degree
- Master’s level degree
- PharmD
- EdD
- MD
- PhD
- Other (please specify: _________)

1. Prior to OPEN did you have experience working with a multi-institutional collaboration?

- Yes
- No

**Appendix B. Interim OPEN Member Survey and Interview: Interview Guide**

1. How long have you been with OPEN?
2. At what site are you located?
3. How would you describe your role in the OPEN program

PROBE: Is it clear to you? / Is this what you expected?

**We have some questions for you about the Gender and Vulnerable populations theme…**

1. What role do you see the GVP team playing within OPEN?
2. How about within your own OPEN research?
   1. How have you considered gender or vulnerable populations dimensions in your research (or research in which you have been involved)?
      1. *If they haven’t*: can you talk about why?
      2. *If they have:* How has this changed since the start of OPEN? How have the GVP materials been most useful (or not been useful)?
   2. How have the OPEN GVP components *specifically* enhanced or impacted your research practice (or research in which you have been involved)?
      1. PROBE: Consider these impacts in relation to research barriers in terms of your knowledge/skills/attitudes.
3. How can the GVP team improve to better meet your needs? (If you could make one change…?)

**Are you a project lead?**

1. **If YES**, Thinking about your project team, how has it functioned within the context of OPEN
   1. How did your team, as a whole, engage with GVP team?
   2. Did you your team have an opportunity to address gaps or deficits in knowledge/skills/attitudes related to their research using the materials/ resources offered by the GVP team?
   3. What are some problems/ challenges/ successes you’ve experienced in regard to your work with GVP? Please be specific.
   4. What are some future opportunities you see to address team members’ skills, knowledge or attitudes by drawing on GVP materials? Please be specific.

**If NO, move on to thanks**

**End-of-Study OPEN Member Survey**

1. Please identify yourself and your team.
2. Name of Team:
3. Name of individual submitting this information:
4. To assist us in reporting how sex/gender was considered and incorporated into your research, please answer the following questions for each of your team's research projects.
5. Project 1 Title:
6. Brief summary of the project's methods:
7. At what stage was sex and/or gender considered in this project? (Please check all that apply.)

- Literature review
- Research questions
- Methodology
- Findings
- Discussion
- KT products
- None of the above
- Other. Please specify ____________________

1. At what stage was sex and/or gender incorporated in this project? (Please check all that apply.)

- Literature review
- Research questions
- Methodology
- Findings
- Discussion
- KT products
- None of the above
- Other. Please specify ____________________

1. What data did you collect or analyze that included sex and/or gender? (e.g., administrative data, surveys, interviews, focus groups, etc.)
2. Did you have published materials that report sex and/or gender findings? (Please give references if available.)
3. Do you have other product outputs (e.g., reports, posters) that report on sex and/or gender? (Please give brief description.)
4. Would you like to report on another research project?

- Yes
- No

1. Did your team use any GVP resources?

- Yes
- No

1. Did your team use the Sex/ Gender and Intersectionality learning module?

- Yes
- No

1. Are there any comments you wish to share about why your team did or did not use the Sex/ Gender and Intersectionality learning module (e.g., use to familiarize team/ students with concepts, did not use module because it was time consuming)?
2. Did your team use the suggested demographic questions?

- Yes. We used the sex/ gender questions only.
- Yes. We used the other questions only.
- Yes. We used both the sex/ gender and other questions.
- No

1. Are there any comments you wish to share about why your team did or did not use the demographic questions?
2. Did your team have any individual consultation with the GVP team? (In-person, phone, email, etc.)

- Never
- Once
- A few times (e.g., 2 -4 times total)
- Often (once every few months)
- Frequently (once every few weeks or more)

1. Are there any comments you wish to share about why your team did or did not have individual consultations with the GVP team?
2. Do you have any questions for the GVP team at this time?
